# Deletion of the GI-2 integrase and the *wbkA* flanking transposase improves the stability of *Brucella melitensis* Rev 1 vaccine

**DOI:** 10.1186/1297-9716-44-105

**Published:** 2013-10-31

**Authors:** Marcos Mancilla, María-Jesús Grilló, María-Jesús de Miguel, Ignacio López-Goñi, Beatriz San-Román, Ana Zabalza-Baranguá, Ignacio Moriyón

**Affiliations:** 1 Departamento de Microbiología y Parasitología e Instituto de Salud Tropical, Universidad de Navarra, C/ Irunlarrea, 1, 31008 Pamplona, Spain; 2 Instituto de Bioquímica y Microbiología, Universidad Austral de Chile, Campus Isla Teja, casilla 567, Valdivia, Chile; 3 Grupo de Sanidad Animal, Instituto de Agrobiotecnología (CSIC-Universidad Pública de Navarra-Gobierno de Navarra), Campus de Arrosadía, 31006 Pamplona, Spain; 4 Unidad de Sanidad Animal, Centro de Investigación y Tecnología Agroalimentaria de Aragón (CITA), Gobierno de Aragón. Av. Montañana, 930, 50059 Zaragoza, Spain

## Abstract

*Brucella melitensis* Rev 1 is the best vaccine available for the prophylaxis of small ruminant brucellosis and, indirectly, for reducing human brucellosis. However, Rev 1 shows anomalously high rates of spontaneous dissociation from smooth (S) to rough (R) bacteria, the latter being inefficacious as vaccines. This S-R instability results from the loss of the O-polysaccharide. To overcome this problem, we investigated whether some recently described mechanisms promoting mutations in O-polysaccharide genes were involved in Rev 1 S-R dissociation. We found that a proportion of Rev 1 R mutants result from genome rearrangements affecting the *wbo* O-polysaccharide loci of genomic island GI-2 and the *wbkA* O-polysaccharide glycosyltransferase gene of the *wbk* region. Accordingly, we mutated the GI-2 *int* gene and the *wbk* IS transposase involved in those arrangements, and found that these Rev 1 mutants maintained the S phenotype and showed lower dissociation levels. Combining these two mutations resulted in a strain (Rev 2) displaying a 95% decrease in dissociation with respect to parental Rev 1 under conditions promoting dissociation. Rev 2 did not differ from Rev 1 in the characteristics used in Rev 1 typing (growth rate, colonial size, reactivity with O-polysaccharide antibodies, phage, dye and antibiotic susceptibility). Moreover, Rev 2 and Rev 1 showed similar attenuation and afforded similar protection in the mouse model of brucellosis vaccines. We conclude that mutations targeting genes and DNA sequences involved in spontaneous O-polysaccharide loss enhance the stability of a critical vaccine phenotype and complement the empirical stabilization precautions taken during S *Brucella* vaccine production.

## Introduction

The members of the genus *Brucella* are gram-negative bacteria that cause brucellosis, an infection affecting domestic and wild animals as well as human beings. The genus includes several species among which *B. melitensis*, *B. abortus* and *B. suis* are the main cause of brucellosis in domestic livestock. *B. melitensis* preferentially infects sheep and goats and represents the most common cause of human brucellosis, a severe and debilitating disease endemic in large areas of Africa, the Middle East, Asia and Latin America [1]. This distribution reflects the problems associated with the control and eradication of a zoonotic disease in areas where extensive breeding and infrastructure weaknesses are combined. Accordingly, the World Health Organization has classified brucellosis as one of the top neglected zoonosis, a group of diseases that hamper development and contribute to the perpetuation of poverty [2].

The most effective measure to control brucellosis is the vaccination of the host ruminants [3]. Several vaccines have been developed for this purpose but the smooth (S) attenuated *B. abortus* S19 and *B. melitensis* Rev 1 strains are superior in controlled experiments [4-6] and the only ones that have proved their usefulness in successful eradication programs. Human-to-human contagion is only anecdotal, and there is abundant evidence showing that the correct use of vaccine Rev 1 results in a parallel decrease of human brucellosis, as illustrated by the experience in Greece [7]. Indeed, the use of a vaccine of good quality is essential and, in this regard, Rev 1 is not lacking problems. It is well know that, as compared to virulent *B. melitensis*, Rev 1 has a marked tendency to undergo the smooth-rough (S-R) dissociation that results in a dominancy of non-immunogenic R mutants that make the vaccine ineffective [8]. Indeed, procedures that minimize the S-R dissociation and controls to exclude batches containing R bacteria are critical in Rev 1 production [8].

**Figure 1:**
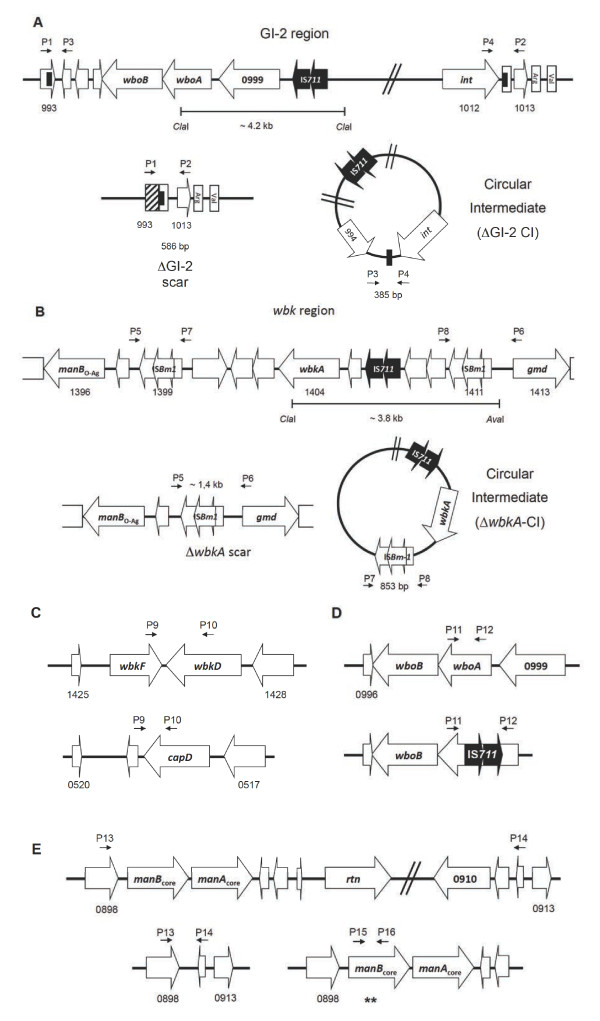
**Genetic organization of regions encoding LPS genes that are susceptible to mutation with a subsequent generation of R mutants. (A)** GI-2 region of *B. melitensis* and the scar and circular intermediate (*wbo* genes are omitted in the circular intermediate) resulting from the *int*- promoted excision; **(B)***wbkA* region and the corresponding scar and circular intermediate resulting from homologous recombination between IS*Bm1* transposases; **(C)** the *wbk*FD region of *B. melitensis* and *B. canis* (wbkD is annotated as capD in B. canis); **(D)***wbo* locus in *B. melitensis* and *B. abortus* RB51 and **(E)***manBA*_core_ locus in *B. melitensis* chromosome II with its corresponding mutants showing the deletion and point mutations in *manB*_core_ gene (asterisks). The position of the primers used in the PCR analyses is indicated as P-number of the primer. The ORF annotations are based on the *B. melitensis* 16 M genome [GenBank: AE008917 and AE008918] and *B. canis* chromosome I [GenBank: CP000872].

In the brucellae, the S-R dissociation is caused by the spontaneous mutations leading to the loss of the O-polysaccharide (O-PS) of the outer membrane lipopolysaccharide (LPS). The LPS O-PS genes are concentrated in two genetic regions: *wbk* (which includes genes of glycosiltransferases, enzymes for the synthesis of precursors and bactoprenol priming, and several insertion sequences [IS]) and *wbo* (carrying the *wboA* and *wboB* glycosyltransferase genes), the latter being part of genomic island 2 (GI-2) [9-12]. Other genes coding for glycosyltransferases and synthesis of precursors are scattered in the genome [9,13]. Although the causes of the S-R dissociation have not been completely elucidated, there have been recent advances in our understanding of the main genetic mechanisms involved. In wild-type *B. abortus*, the spontaneous excision of 15.1 kb GI-2 island (which removes the *wboA* and *wboB* loci) by recombination promoted by the GI-2-encoded integrase (*int*) is one of the main sources of S-R dissociation, and identical dissociation mechanisms have been proposed for *B. melitensis* and *B. suis* (Figure 1A) [10]. A similar spontaneous recombination and excision affects *wbkA* (a *wbk* glycosyltransferase gene), in this case mediated by RecA, which recognizes the flanking *wbkA* transposases as substrates for homologous recombination [14] (Figure 1B). In both cases, the resulting R mutants carry a chromosomal scar that results from the release of a circular intermediate (Figure 1A and 1B) that is lost in subsequent rounds of replication. In addition, a proportion of R mutants result from random mutations affecting the *manB*_core_ gene and possibly other LPS genes related to the synthesis of O-PS and core oligosaccharide precursors and their assembly [15]. The R phenotype of the *B. abortus* RB51 vaccine is also known to relate in part to the disruption of gene *wboA* of GI-2 by IS711 (Figure 1D), which indicates the mobility of IS*711* as a source of R mutations [16]. Examination of the LPS genes of *B. canis* and *B. ovis*, two naturally R species, confirms some of the above mechanisms and reveals additional possibilities for the generation of defective LPS. *B. canis* carries a deletion encompassing genes *wbkD* and *wbkF* (Figure 1C) probably generated by a slipped mispairing mechanism [17]. *B. ovis* lacks GI-2 (and bears the corresponding genomic scar) and carries mutations affecting *manB*_core_, *wbkF* and other *wbk* genes (Figure 1E) [10-12,15,17].

Since GI-2 and region *wbk* are conserved in S brucellae, we hypothesized that GI-2 and *wbkA* excisions could be involved in the S-R dissociation of vaccine Rev 1, and that the stability of this vaccine could be improved. Here we show that these two mechanisms account for the majority of the mutations involved in the S-R dissociation of this vaccine. We also show that mutation of the *int* gene in GI-2 and the IS-sequences flanking *wbkA* in Rev 1 stabilizes the S phenotype without altering the biologically relevant characteristics of the vaccine*.* Accordingly, we propose the corresponding double GI-2 *int* and *wbkA* IS mutant of Rev 1 as an improved, more stable vaccine against *B. melitensis* infection in small ruminants.

## Materials and methods

### Bacterial strains and culture conditions

The *Brucella* strains used are listed in Table 1, and the *E. coli* strains and plasmids are in Additional file 1. For S-R dissociation experiments, three lots of commercial Rev 1 vaccine showing 0%, 5% and 100% of dissociation (determined by the crystal violet exclusion method, following the standard protocol for vaccine quality control [8]) were selected from a collection kept in the Centro de Investigación y Tecnología Agroalimentaria of Aragón (CITA), Zaragoza, Spain. Bacteria were grown either on trypticase soy agar (TSA; Becton Dickinson, Madrid, Spain), Blood Agar Base number 2 (BAB; Oxoid, Hampshire, UK) plates (37 °C, 1–5 days) or in trypticase soy broth (TSB; Becton Dickinson, Madrid, Spain). For cloning, *E. coli* was grown in Luria-Bertani broth (LB; Becton Dickinson, Madrid, Spain) supplemented with kanamycin (Km; 50 μg/mL) or chloramphenicol (Ch; 20 μg/mL). Subcultures in BAB supplemented with nalidixic acid (Nx; 25 μg/mL) were used for obtaining the *B. melitensis* H38-Nx^R^ spontaneous variant used as challenge strain in vaccine experiments in mice.

**Table 1:** ***Brucella *** **strains used.**

**Strain**	**Characteristics**	**Source/ reference**
*B. melitensis* H38	*B. melitensis* smooth virulent strain; S-LPS	CITA collection
H38-Nx^R^	*B. melitensis* H38 spontaneous mutant resistant to nalidixic acid; challenge studies in mice; S-LPS	This work
Rev 1	*B. melitensis* smooth attenuated reference vaccine strain; S-LPS	CITA collection
Rev 1Δ*wbkA*	Rev 1 spontaneous deletion mutant in *wbkA* (ORF BMEI1398 and BME1412); R-LPS	This work
Rev 1ΔGI-2	Rev 1 spontaneous deletion mutant in GI-2; R-LPS	This work
Rev 1ΔIS*Bm1*	Rev 1 in-frame deletion mutant in IS*Bm-1* transposase gene (ORF BMEI1398 and BME1400); S-LPS	This work
Rev 1Δ*int*	Rev 1 in-frame deletion mutant in GI-2 phage-integrase gene (nucleotides *int*_Δ53–286_); S-LPS	This work
Rev 2(ΔIS*Bm1*Δ*int*)	Rev 1 double in-frame deletion mutant in IS*Bm-1* transposase and GI-2 phage-integrase; S-LPS	This work
Rev 1Δ*wbkA*/c	Rev 1Δ*wbkA* rough spontaneous mutant complemented with plasmid pMM14; S-LPS	This work
Rev 1ΔGI-2/*wboAB*	Rev 1ΔGI-2 rough spontaneous mutant containing the plasmid pMM76; R-LPS	This work
Rev 1ΔGI-2/c	Rev 1ΔGI-2 rough spontaneous mutant complemented with plasmid pBGI-997-99c; S-LPS	This work

S-LPS: smooth lipopolysaccharide; CITA: Centro de Investigación y Tecnología Agroalimentaria of Aragón (Zaragoza, Spain); R-LPS: rough lipopolysaccharide; GI-2: Genomic Island 2.

### Sequence analyses

Confirmation of constructed mutant genotypes and analysis of *manB*_core_ locus in spontaneous R mutants was carried out on PCR fragments by DNA sequencing, using the dideoxy method at the Sequencing Unit of Centro de Investigación Médica Aplicada (CIMA, Universidad de Navarra, Spain). In silico mutants were designed using the complete sequence of chromosome I of *B. melitensis* 16 M as template [GenBank: AE008917] [18]. Similarity searches were performed by BLAST [19] and sequence alignments by Clustal Omega [20]. In silico restriction analyses of *wbo* and *wbk* regions were performed with Vector NTI software (Invitrogen, USA).

### PCR assays

The primers designed to identify mutations known to affect the O-PS generating R phenotypes are indicated in Figure 1 and listed in Table 2. In addition, primers BMEI0993F and BMEI1013R (GI-2 deletion) or BMEI1398F and BMEI1413R (*wbkA* deletion) were used for detecting chromosomal scars, and primers BMEI0994R and BMEI1012bF (GI-2 region) or BMEI1400R and BMEI1409F (*wbkA* gene) were used for detecting the circular intermediates. Primers were generated with Primer3 software [21]. Genomic DNA (gDNA) was obtained by using standard protocols [22] or boiling (95 °C, 10 min) either directly from bacterial colonies grown in TSA plates or from Rev 1 commercial vaccine vials rehydrated in saline. PCR assays were performed in a final volume of 25 μL containing 0.2 μg of gDNA, 12.5 pmol of the correspondent primer, 0.2 mM of dNTPs, 2 mM of MgCl_2_ and 1U of Immolase DNA polymerase (Bioline, London, UK). The mixture was pre-incubated at 95 °C for 5 min, followed by 30 cycles (95 °C for 20 s; 60 °C for 30 s; and 72 °C for 1 min) with a final extension at 72 °C for 5 min. All PCR rounds were carried out in a Mastercycler gradient PCR machine (Eppendorf, Hamburg, Germany). Amplicons and restriction fragments were resolved by electrophoresis in 1.0-2.0% TBE (45 mM Tris-borate, 1 mM EDTA pH 8.0) agarose gels. All PCR experiments included DNA from S Rev 1 as a control.

**Table 2:** List of oligonucleotides used.

**Name**	**Sequence (5′→ 3′)**	**Name in Figure**1	**Reference**
711u	cacaagactgcgttgccgacaga		[23]
711d	catatgatgggaccaaacacctaggg		[23]
BMEI0999F	cacgatcaaaacgatgccct		This study
BMEI0999R	ccaaaatgtcctgagcttgg		This study
BMEI1404F	aagggctggaacctaggaga		This study
BMEI1404R	aatgacttccgctgccatag		This study
BMEI0993F	caacatcgcaaagcctgaaa	P1	[10]
BMEI1013R	cgcaatccagccaatacctg	P2	[10]
BMEI0994R	atcgtcggcattgtctctct	P3	[10]
BMEI1012bF	attatccggcggtatgtgag	P4	[10]
BMEI1398F	gatcttggtatcggcctgtc	P5	[14]
BMEI1413R	tgcgactttcttcacgattg	P6	[14]
BMEI1400R	cgctttaatatctcgcgttcc	P7	[14]
BMEI1409F	ggtcccatcggcatatctt	P8	[14]
BMEI1426F	ctggagtgtgccgaaagtg	P9	This study
BMEI1427R	gctgatctcttccgacaagg	P10	This study
BMEI0998F	ttaagcgctgatgccatttccttcac	P11	This study
BMEI0998R	gccaaccaacccaaatgctcacaa	P12	This study
BMEII0898F	tcggcacagcaagctataaa	P13	This study
BMEII0912R	ggtgtggatattgcgctttc	P14	This study
BMEII0899F	ccgcctatgcctatacgatg	P15	This study
BMEII0899R	gcctcatcatccttgtcgat	P16	This study
BMEI1401_F1	ttctcgagagcctgaagagc		This study
BMEI1401_R2	gccttcgtcgagaaaatgag		This study
BMEI1397_F3	ctcattttctcgacgaaggccgtttgcatcaatcagttcg		This study
BMEI1397_R4	ctcggctggcagtatctttc		This study
BMEI1012_F1	caaagagctaagggcattcg		This study
BMEI1012_R2	cgcgaaactttgaagcatct		This study
BMEI1012_F3	agatgcttcaaagtttcgcgtctatatcgccggtctgtcc		This study
BMEI1012_R4	tttcagtgctttatgacgaaaat		This study

*F*, forward; *R*, reverse.

### Southern blot

The distribution of IS*711* sequences was assessed by Southern blot using 1–2 μg of *Ava*I-*Cla*I double digested gDNA from *B. melitensis* Rev 1 related-strains as previously described [23]. Restriction fragments were resolved in 1% agarose-TBE at 25 mA for 10 h and probed with ECL direct-labeled system (GE Healthcare, Waukesha, WI, USA). IS*711* fragment was generated by PCR with primers 711u and 711d as well as the corresponding GI-2 (~1.4 kb, BMEI0999 primers) and *wbkA* (332 bp, BMEI1404 primers) specific DNA probes (Table 2). Chemiluminiscent detection of the hybridized product was performed using a commercial kit (GE Healthcare, Madrid, Spain) and enhanced ECL Films for developing by conventional photographic methods. Blots were stripped with 1% SDS at 80 °C for 30 min and EDTA 20 mM at room temperature for 15 min, and then analyzed for the presence of GI-2 and *wbkA* with specific DNA probes.

### Mutagenesis

The Rev 1 strain used in these experiments was a vaccine seed lot of proved efficacy. It was generously provided by Dr J.M. Blasco (CITA of Aragón, Spain). For mutagenesis, a parental Rev 1 clone was selected directly from colonies grown on agar according to morphology, size and agglutination criteria previously described [24,25]. A first Rev 1 (ΔIS*Bm1* strain) mutant carrying a deletion of 872 bp spanning ORFs BMEI1398-1400 (IS*Bm1* complete transposase) was constructed by allelic exchange [26]. Briefly, PCR fragments produced with primers pairs BMEI1401_F1, R2 and BMEI1397_F3, R4 (Table 2) were ligated by PCR overlap, and the resulting fragment was cloned into pCR2.1 TOPO (Invitrogen, USA) to produce plasmid pMM20. The *Bam*HI-*Not*I fragment of pMM20 was subcloned into plasmid pJQK [27] to generate the pMM22 suicide vector, which was transferred to Rev 1 by conjugation using *E. coli* S17-1λ*pir*[28]. Double crossover transconjugants were selected on polymyxin 1.5 μg/mL and sucrose 5% plates, and the mutants were identified by PCR. The same strategy was used to construct a Rev 1Δ*int* (BMEI1012) non-polar mutant. Two fragments generated with primer pairs BMEI1012_F1, R2 and BMEI1012_F3, R4 (Table 2) were ligated by PCR. The resulting fragment containing a copy of *int* lacking the nucleotides encoding amino acids 53–286 was cloned into pCR2.1 TOPO to produce the plasmid pMM55. Then, the *Bam*HI-*Xba*I fragment of pMM55 was subcloned into the plasmid pJQK to generate the pMM56 suicide vector, which in turn was transferred to Rev 1 by conjugation, and the mutants were identified by PCR. Mutant Rev 1 ΔIS*Bm1* was used to construct the double mutant ΔIS*Bm1*Δ*int* (hereafter Rev 2) using the mutator plasmid pMM56. Maintenance of the reading frame was verified by sequencing of suicide vectors and of PCR amplified fragments of the final constructs [26].

### Complementation

To confirm the origin of the mutation, spontaneous Rev 1 R strains lacking *wbkA* were complemented with plasmid pMM14, which carries a copy of *wbkA*[14]. In the case of spontaneous ΔGI-2 Rev1 R mutants, plasmids pMM76 (carrying *wboA*-*wboB*) or pBGI-997-99c (carrying *wboA*-*wboB* plus ORF BMEI0999) were used [29].

### S-R dissociation assessment

A loop of bacteria previously grown on TSA plates for 72–96 h at 37 °C was transferred to a flask containing 10 mL of TSB, and the flask was incubated until bacteria reached the stationary phase (37 °C, 72–96 h). An aliquot of this culture was adjusted to 0.109 of optical density at 750 nm (equivalent to 10^8^ CFU/mL) using sterile TSB and then diluted 1:100 in the same broth. After incubation for 3–4 days at 37 °C, serial ten-fold dilutions were plated on TSA, plates were incubated (37 °C, 5 days), and S-R dissociation was assessed by the crystal violet exclusion method and the R phenotypes confirmed by agglutination with 0.1% acriflavine [5,8]. Dissociation rate was calculated as the ratio between the number of R colonies and the total colony counts. In addition, commercial Rev 1 vaccine lots were analyzed for dissociation by PCR amplification (see above) of ∆GI-2 and ∆*wbkA* chromosomal scars using DNA obtained directly from vaccine batches reconstituted as recommended by the manufacturers.

### Bacteriological characterization

*Brucella* spp. and Rev 1-specific phenotypic characteristics were confirmed or assessed by established procedures [24,25]. In addition, growth curves were obtained in a BioScreen C [30] apparatus. For this, the parental Rev 1, the Rev 2 double mutant ΔIS*Bm1*Δ*int* and the virulent *B. melitensis* H38 (as a control) were inoculated (5 × 10^6^ CFU/well) by triplicate in Bioscreen multi-well plates containing 250 μL/well of TSB, and the optical density was read at 600 nm wavelength (O.D._600_) at 10 min intervals, until to reach the stationary phase.

### LPS characterization

The S or R nature of LPS of the mutant and complemented strains was analyzed by Western blot of SDS-proteinase K extracts using the serum of a rabbit immunized with *B. melitensis* 16 M [9,31]. Purified S and R-LPS obtained as described previously [9,31] were used as controls.

### Experiments in mice

The biological properties of Rev 1ΔIS*Bm1*Δ*int* mutant (Rev 2) determined in comparison with the reference Rev 1 vaccine were virulence and protective efficacy in the mouse model. Female BALB/c mice 7–8 weeks old were purchased from Charles River International (France) and accommodated in the animal facilities of CITA (registration code ES/50-2970-12005) or Universidad Pública de Navarra (UPNA; registration code ES/31-2016-000002 CR-SU-US) for 1–2 weeks before starting and during the assays, with water and food *ad libitum*. Animal handling was performed in compliance with current European and national (RD 53/2013) regulations, following the FELASA and ARRIVE guidelines, and with the approval of the CITA or UPNA Animal Experimentation Committees and local Government authorization (approval reference number R130/2012). Mice inoculations were carried out with 0.1 mL of bacterial suspension previously adjusted to an optical density at 600 nm of 0.170 (approximately 1 × 10^9^ CFU/mL) and then diluted to the appropriate dose (see below) in sterile phosphate buffered saline [32]. For virulence, groups of 30 mice each were inoculated intraperitoneally with 1 × 10^5^ CFU/mouse of Rev 2 or Rev 1 attenuated strains. As a control, an additional group of 30 mice were inoculated similarly with the virulent *B. melitensis* H38 reference strain. Spleen weights and viable counts (*n* = 5) were determined at 1, 3, 6, 9, 12 and 15 weeks post-inoculation as described previously [32]. The identity of the spleen isolates was confirmed by PCR, and the constancy of the typical Rev 1 phenotypic features confirmed throughout the experiments. Spleen weights were expressed as the mean and SD (*n* = 5) of grams/spleen and the level of spleen infections as mean ± SD (*n* = 5) of individual log_10_ CFU/spleen at the indicated times.

Efficacy studies were carried out in groups of 5 BALB/c mice each vaccinated subcutaneously with 1 × 10^5^ CFU/mouse of Rev 2 or Rev 1 (parental control), or with 0.1 mL of phosphate buffered saline (pH 6.85) as the placebo vaccinated control. Four weeks after vaccination, all mice were challenged intraperitoneally with 1 × 10^4^ CFU/mouse of *B. melitensis* H38-Nx^R^, and the number of challenge bacteria in spleens was determined 2 weeks afterwards [32]. Differentiation between challenge and residual vaccine bacteria was performed by double plating on BAB and BAB supplemented with Nx 25 μg/mL. The results were expressed as the mean and SD (*n* = 5) of the log_10_ of *B. melitensis* H38-Nx^R^/spleen. In a previous work, the virulence in mice of the *B. melitensis* H38-Nx^R^ challenge strain used in this work was found to be indistinguishable from that of the reference H38 and 16 M strains [9].

In both virulence and protection experiments, statistical comparisons of means were performed by a one-way ANOVA followed by the Fisher’s Protected Least Significant Differences tests [32].

## Results

### Deletions involving GI-2 and *wbkA* are the major causes of S-R dissociation of *B. melitensis* Rev 1

As a first approach to determine the dissociation mechanisms operating in Rev 1, we searched for ∆GI-2 and ∆*wbkA* chromosomal scars, for deletions in *wbkF* and *wbkD*, insertion of IS elements on *wboA* and *manB*_core_ mutations (Figure 1) in Rev 1 spontaneous R mutants. To this end, we studied a collection of 32 R Rev 1 isolates (previously obtained by repeated plating on TSA and crystal violet and acriflavine agglutination screening) and characterized them by PCR using the primers described in Table 1. We found 8 ΔGI-2 and 3 Δ*wbkA* mutants, but we did not detect redundancy or other mutations known to generate R phenotypes in *Brucella*, including point mutations in *manB*_core_ in the remaining 21 mutants.

**Figure 2:**
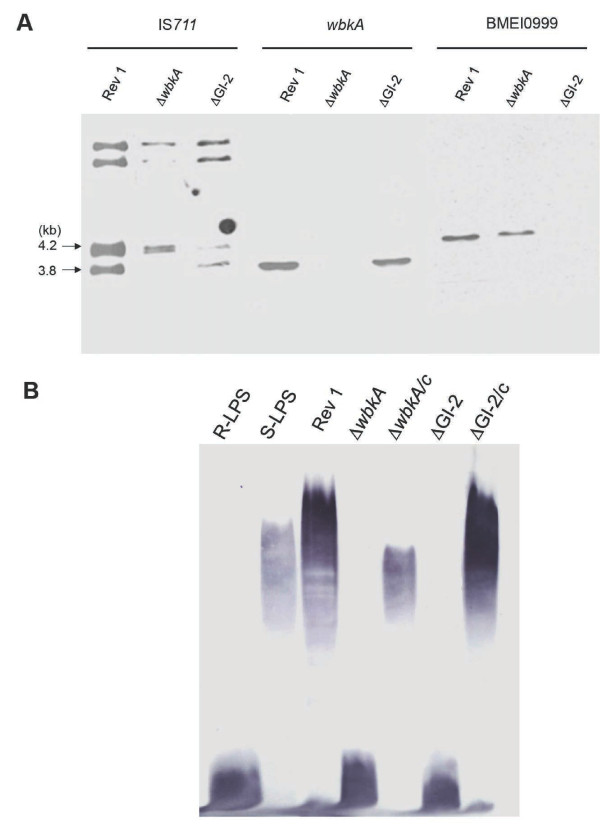
**Genetic and phenotypic characterization of spontaneous Rev 1 R mutants. (A)** Southern blot analysis of *B. melitensis* Rev 1 spontaneous R variants with IS*711* (left panel), *wbkA* (center panel) and GI2 BMEI0999 (right panel) as probes. Note that, for the IS711 probe and *B. melitensis* Rev 1 and ∆*wbkA* but not ∆GI-2*,* the signal at 4.2 kb corresponds to two bands (predicted MW 4.2 and 4.14 kb). For simplicity, fragments with molecular masses lower than 3.8 kb were omitted. **(B)** Western blot of purified R-LPS and S-LPS, Rev 1, *wbkA* and GI-2 R mutants and the corresponding complemented strains probed with anti-LPS antibodies.

To confirm that the above observations corresponded in fact to the expected GI-2 or *wbkA* deletions and O-PS defects, we first characterized Rev 1 R mutants by IS*711*-fingerprinting using DNA probes specific for the *wbkA* and GI-2 loci. We found that, as predicted, ΔGI-2 and Δ*wbkA* Rev 1 mutants lacked the IS*711* bands of 4.2 and 3.8 kb, respectively (Figure 2A). Second, we examined the LPS phenotype by Western blot. Figure 2B shows that the LPS of the Rev 1 mutants lacked the O-PS. Third, we performed complementation experiments in eleven R mutants using plasmids encoding the *wbkA* (pMM14) or the tandem *wboA*-*wboB* of GI-2 (pMM76). We found that the 3 spontaneous Rev 1 Δ*wbkA* R isolates were complemented with plasmid pMM14 (a representative result can be seen in Figure 2B, lane Δ*wbkA*/c). However, we repeatedly failed to complement the Rev 1 ΔGI-2 R isolates with plasmid pMM76 (not shown). Rajashekara et al. [29] demonstrated that artificially constructed *B. melitensis* ΔGI-2 R mutants could be reverted to S phenotype with a plasmid (pBGI-997-99c) bearing *wboA* and *wboB* plus ORF BMEI0999. Using this approach, we could revert 8 spontaneous Rev 1 ΔGI-2 R isolates (a representative result can be seen in Figure 2B, lane ΔGI-2/c). These results show that none of these genetically well-characterized R isolates carried more than one R-linked mutation and, in addition, support the hypothesis that the protein encoded by BMEI0999 plays a role in O-PS synthesis. In summary, the results showed that two previously described dissociation mechanisms, *int*-dependent (for GI-2 excision) and *wbk* IS-RecA dependent (for *wbkA*), are actually active in Rev 1.

Finally, to study whether ∆GI-2 and ∆*wbkA* excisions occurred during vaccine manufacturing, we examined three commercial Rev 1 lots with different levels of dissociation (see Material and methods). We identified the GI-2 and *wbkA* chromosomal scars in these commercial preparations (not shown) and, interestingly, the lot with the highest dissociation rate (almost 100%) presented both chromosomal scars. Considering these results and those of the above-described experiments, we concluded that GI-2 and *wbkA* deletions occur repeatedly in batches during manufacturing despite the precautions taken to minimize S-R dissociation.

### The S-R dissociation of *B. melitensis* Rev 1 is reduced by mutation of GI-2 *int* and IS*Bm1* transposase flanking *wbkA*

**Figure 3:**
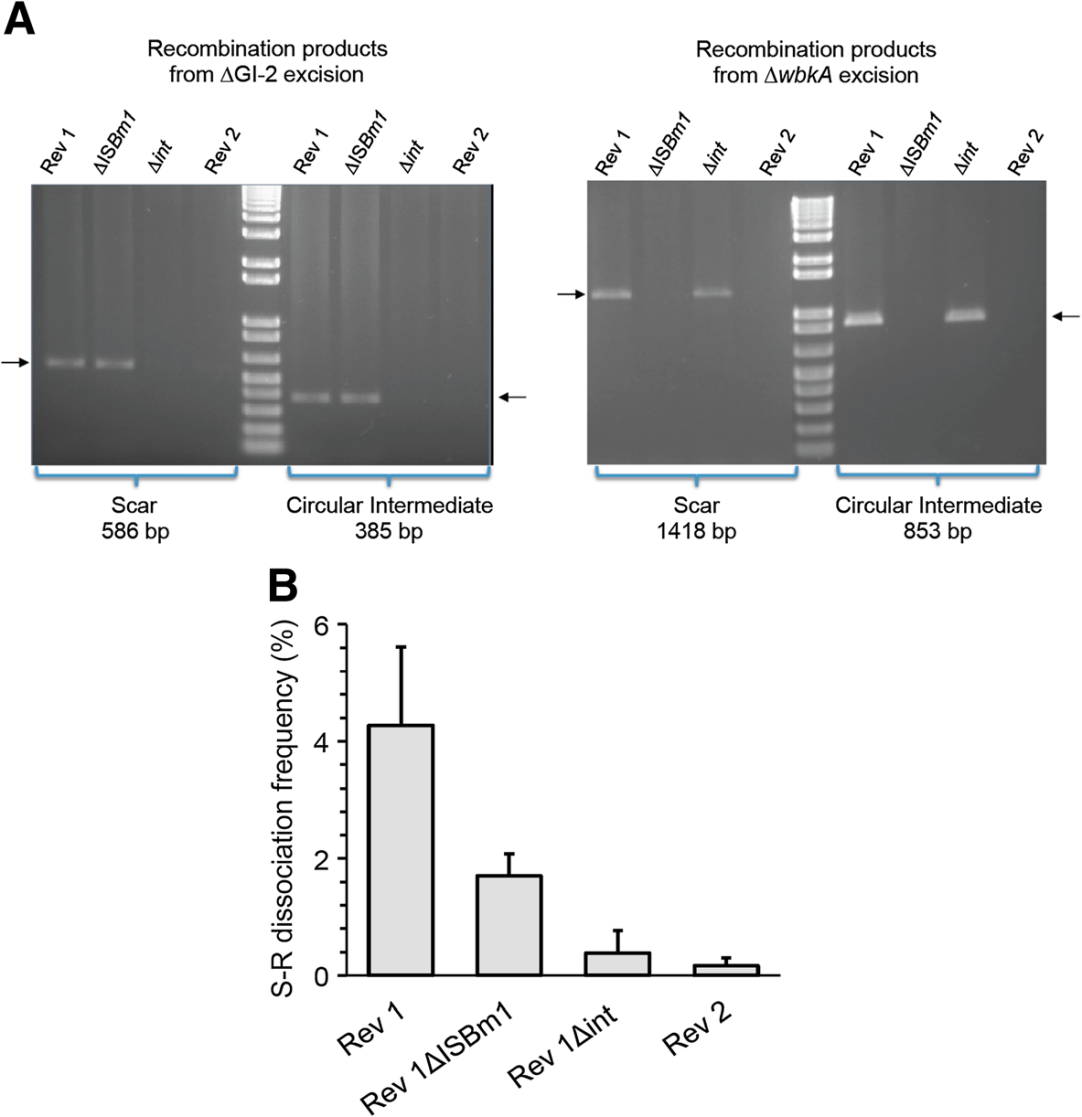
**Stabilization of regions GI-2 and wbkA by deletion of appropriate section of GI-2 phage-related integrase (Δ*int*), *wbkA*-flanking IS (Δ*ISBm1*) and both (Rev 2). (A)** PCR detection of the chromosomal scar and circular intermediate resulting from (GI-2 excision (left panel) and *wbkA* excision (right panel). **(B)** S-R dissociation rates.

We have previously shown that disruption of GI-2 phage-related integrase *int* gene reduces the dissociation rate of *B. abortus* by stabilization of GI-2 [10]. To test whether this also occurs in *B. melitensis* Rev 1, we constructed mutant Rev 1Δ*int* by in-frame deletion of GI-2 *int* (see Material and Methods). Similarly, we have previously shown that the excision of the 5.5 kb fragment carrying *wbkA* is caused by homologous recombination mediated by RecA and the *wbkA*-flanking IS [14]. Thus, we also constructed mutant Rev 1ΔIS*Bm1*, which lacks one of the *wbkA*-flanking IS*Bm1* copies. Characterization by PCR demonstrated only the scar and circular intermediate corresponding to GI-2 excision in Rev 1ΔIS*Bm1* (Figure 3A), showing the stabilization of *wbkA*. Conversely, Rev 1Δ*int* displayed the scar and circular intermediate corresponding to an excision of the *wbkA* (Figure 3A). To stabilize both genetic regions, we constructed the Rev 1ΔIS*Bm1*Δ*int* double mutant (Rev 2) on the Rev 1ΔIS*Bm1* background. As expected, Rev 2 did not show evidence (scars or circular intermediates) of GI-2 or *wbkA* excision events by PCR (Figure 3A) even in experiments with high amounts of template DNA.

Using the above mutants and the parental Rev 1 and *B. melitensis* H38 as references, we assessed the importance of the corresponding genetic mechanisms in the frequency of the S-R dissociation of Rev 1. To this end, we used conditions that favor the growth of R mutants and counted the proportions of R bacteria using the crystal violet exclusion method (see Material and methods, S-R dissociation assessment). While dissociation of strain H38 was beyond detection under the experimental conditions, Rev 1 showed dissociation rates of 4.27 ± 1.34% (Figure 3B). In contrast, the single mutants displayed lower (*p* < 0.0001) dissociation rates than Rev 1. These dissociation rates were significantly (*p* < 0.01) lower for Rev 1∆*int* (0.38 ± 0.33) than for Rev 1ΔIS*Bm1* (1.70 ± 0.38). Consistent with the prediction, the double mutant Rev 2 showed the lowest dissociation rates (0.17 ± 0.13%). Therefore, under these experimental conditions, GI-2 and *wbkA* stabilization resulted in an approximately 25-fold (i.e. about 95%) decrease in dissociation.

### Stabilization of the S phenotype does not alter the properties of Rev 1 in vitro or in mice

**Figure 4:**
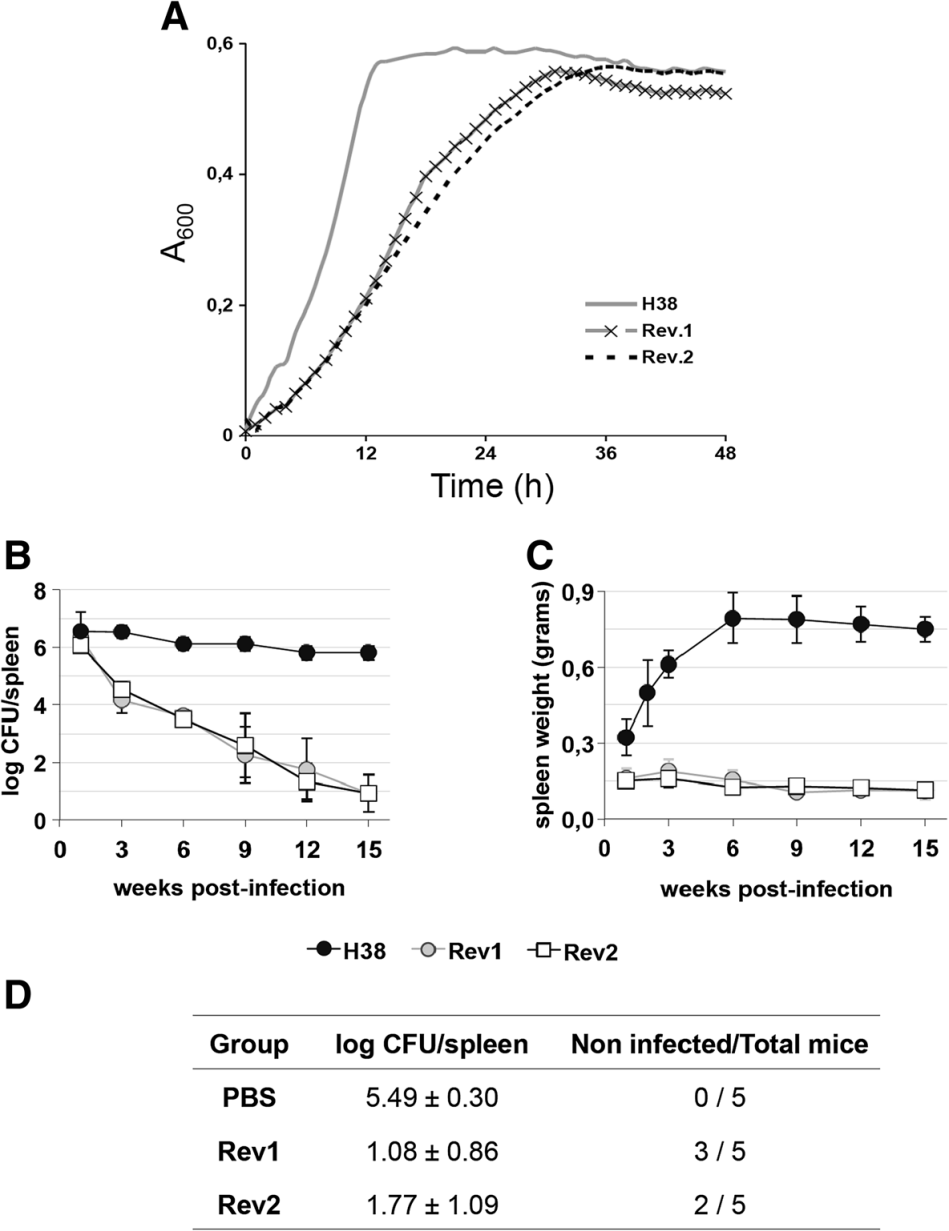
**Genetic stabilization in Rev 2 does not alter the in vitro growth and vaccine properties of Rev 1 reference vaccine. (A)** In vitro growth kinetics; **(B)** Residual virulence (log CFU/spleen); **(C)** Spleen weights; and **(D)** Efficacy against a virulent *B. melitensis* virulent challenge, in BALB/c mice. Statistical comparison of means was performed by one-way ANOVA and Fisher’s PLSD test: ^a^*P* < 0.005 vs. unvaccinated control (PBS group).

Standard bacteriological characterization demonstrated that Rev 1ΔIS*Bm1,* Rev 1Δ*int* and Rev 2 conserved the phenotypic markers that are characteristic of Rev 1, namely: S phenotype (demonstrated by reactivity with anti-S antibodies and by the crystal violet and acriflavine tests), inhibition by 5 IU/mL penicillin, resistance to 2.5 μg/mL of streptomycin, and comparatively small colony size (1–1.2 mm diameter after 5 days of incubation at 37 °C) [24,25]. Since the latter feature (i.e. slow growth) is a particularly important Rev 1 property ([33]; see also below), we compared the growth curves of Rev 1, Rev 2 and the wild-type *B. melitensis* H38 reference strain. As can be seen in Figure 4A, Rev 1 and Rev 2 produced almost identical growth curves and both were retarded with respect to *B. melitensis* H38.

We completed the characterization of Rev 2 by studying whether the critical biological properties of Rev 1 (i.e. residual virulence and efficacy against virulent infection) were maintained in Rev 2. For this purpose, we used the established mouse model for brucellosis vaccine control [34], which is useful in discriminating Rev 1 vaccine lots of inappropriate residual virulence and immunogenicity [25]. Rev 2 showed a behavior almost identical to that of Rev 1 in this model. In virulence assays, both strains persisted similarly and less than the virulent *B. melitensis* H38 strain (Figure 4B). Likewise, Rev 1 and Rev 2 induced a similarly reduced splenomegaly (Figure 4C). Finally, Rev 2 was as protective as Rev 1 against a challenge infection with the full-virulent *B. melitensis* H38-Nx^R^ strain (Figure 4D).

## Discussion

A major practical aspect of vaccine production is quality control. Because of the well-known genetic instability of all brucellae in vitro [34], control is particularly critical in brucellosis vaccines. Therefore, a manufacturing seed-lot system [33] has been adopted to reduce genetic drifts including the appearance of R types. In addition, control of every single batch includes the assessment of colony S phase and morphology [24]. As confirmed here by comparison with *B. melitensis* H38, dissociation of *B. melitensis* Rev 1 into S and R forms is particularly troublesome and, although the reasons for this difference between the virulent bacteria and the vaccine are not known, it is possible to indicate probable causes. Our results indicate that the genetic mechanisms operating in Rev 1 dissociation are those described before for other S strains and, therefore, it seems unlikely that this Rev 1 trait follows particular genetic characteristics of this vaccine. A more probable possibility is suggested by the well-known fact that oxygen limitation and other conditions causing energy shortage facilitate the establishment of *Brucella* R variants [35]. Accordingly, we have proposed [14] that mutants not synthesizing the costly O-PS are more competitive when energy shortage and no selective pressure to maintain the O-PS concur and that, under these circumstances, the excision of GI-2 promoted by the phage-related integrase *int* gene and the deletion of *wbkA* by homologous recombination become prominent. In the case of Rev 1, it is known that this vaccine carries a defect in ribosomal protein S12 that relates to its streptomycin resistance [36]. In *Salmonella* Typhimurium, a similar S12 mutation causes streptomycin resistance, increased rates of ribosomal proofreading and, as a result, decreased rates of protein synthesis, reduced bacterial growth and attenuation [37]. Therefore, it seems likely that the S12 defect in Rev 1 is a major cause of reduced fitness and growth rates (and attenuation), both of which could be compensated for by the reduction in energy expenditure caused by at least (see below) the loss of O-PS, thereby favoring the growth of R mutants. In fact, Rev 1 is characterized by a small colony size and the S-R dissociation is often accompanied by the appearance of larger colonies not appropriate for vaccine use [25].

Although we investigated other known R-generating mutations, we only detected the GI-2 and *wbkA* deletions in Rev 1. Turse et al. [15] have shown that point mutations on *manB*_core_, a gene required for the complete synthesis of O-PS [9], are a cause of spontaneous appearance of R variants, which have been isolated from infected macrophages and mice [15]. We failed to reveal any point mutations in this locus by sequencing *manB*_core_ in the 21 R strains that did not show either *wbkA* or GI-2 deletions. Since Turse et al. performed their experiments with *B. abortus* 2308 and *B. melitensis* 16 M and recovered the R mutants using agar plates saturated with Bk2 phage (specific for the S-phase), it seems that the discrepancies could be explained by the use of different experimental conditions. Concerning the genetic mechanisms, we observed that inactivation of the GI-2 integrase had a more significant effect on O-PS stability. This may reflect a higher instability of this region related to the activity of the integrase and/or to the concomitant loss of Omp25b (a major outer membrane protein encoded in GI-2 [11]), since the ensuing reduction in biosynthetic expenditures would increase competitiveness in vitro. Another possibility is suggested by the fact that recombinases like the one coded for by GI-2 *int* are able to identify secondary target repeat sequences promoting the excision of unrelated fragments [38]. Therefore, the GI-2 integrase could theoretically take part in other events leading to the R phenotype. This work also shows that mutations other than those described thus far also cause the appearance of R types. This is shown by the fact that dissociation was not completely prevented in Rev 2 strain. Further investigations are necessary for a full understanding of the mutation paths that affect the O-PS in *Brucella*.

Because the underlying mechanisms were known, we could abrogate the GI-2 and *wbkA* deletions and generate a more stable Rev 2 strain. Indeed, as shown by the experimental results presented here, the absence of large sections of the DNA sequences involved in this deletion events make highly unlikely the possibility that Rev 2 could revert to an anomalously high S-R dissociation rate. Thus, the genetic strategy described here represents a complement to the empirical methods currently used in the production of smooth *Brucella* vaccines for the stabilization of smooth phenotype. Although the comparison between the virulent H38 strain and Rev 1 showed differences in both growth and S-R dissociation rates, our results demonstrate that the increased S-R dissociation is not linked to attenuation of Rev 1 in the mouse model, as it might be expected from the characteristic attenuation of *Brucella* R mutants. As discussed above, the reduced fitness probably caused by the S12 mutation that is manifested by the lower growth rates is a more likely cause of attenuation. Rev 2 conserved this characteristic and also the relevant biological properties of Rev 1 in the mouse model. Although this model represents the best laboratory control for the in vivo properties of Rev 1 [33], experiments in the natural hosts are clearly necessary to prove that Rev 2 retains all the properties that have made Rev 1 a succesful vaccine.

## Competing interests

The authors declared that they have no competing interests.

## Authors’ contributions

MM designed and performed the molecular biology experiments. MJdM, AZB and BSR carried out the bacteriological studies and experiments in mice. ILG provided financial support. IM, MJG and MM designed the work and wrote the manuscript. All authors read and approved the final manuscript.

## Supplementary Material

Additional file 1 ***E. coli* strains and plasmids used.** List, characteristics and source/reference of *E. coli* strains and plasmids used. Click here for file

## References

[B1] Grace D Mutua F Ocungo P Kruska R Jones K Brierley L Lapar L Said M Herrero M Duc Phuc P Elich Tao N Akuku I Ogutu F Mapping of poverty and likely zoonoses hotspots Zoonoses Project 2012 4 1 119

[B2] Anonymous The control of Neglected Zoonoses Diseases: A route to poverty alleviation 2006 Geneva: WHO Press

[B3] Blasco JM A review of the use of *B. melitensis* Rev 1 vaccine in adult sheep and goats Prev Vet Med 1997 31 275 283 10.1016/S0167-5877(96)01110-5 9234451

[B4] Moriyón I Grilló MJ Monreal D González D Marín C Lopez-Goñi I Mainar-Jaime RC Moreno E Blasco JM Rough vaccines in animal brucellosis: structural and genetic basis and present status Vet Res 2004 35 1 38 10.1051/vetres:2003037 15099501

[B5] Barrio MB Grilló MJ Muñoz PM Jacques I González D De-Miguel MJ Marín CM Barberan M Letesson JJ Gorvel JP Moriyón I Blasco JM Zygmunt MS Rough mutants defective in core and O-polysaccharide synthesis and export induce antibodies reacting in an indirect ELISA with smooth lipopolysaccharide and are less effective than Rev 1 vaccine against *Brucella melitensis* infection of sheep Vaccine 2009 27 1741 1749 10.1016/j.vaccine.2009.01.025 19186196

[B6] Godfroid J Scholz HC Barbier T Nicolas C Wattiau P Fretin D Whatmore AM Cloeckaert A Blasco JM Moriyón I Saegerman C Muma JB Al-Dahouk S Neubauer H Letesson JJ Brucellosis at the animal/ecosystem/human interface at the beginning of the 21st century Prev Vet Med 2011 102 118 131 10.1016/j.prevetmed.2011.04.007 21571380

[B7] Minas A Minas M Stournara A Tselepidis S The “effects” of Rev-1 vaccination of sheep and goats on human brucellosis in Greece Prev Vet Med 2004 64 41 47 10.1016/j.prevetmed.2004.03.007 15219968

[B8] Alton GG Jones LM Angus RD Verger JM Techniques for the brucellosis laboratory 1988 Paris, France: INRA

[B9] González D Grilló MJ De-Miguel MJ Ali T Arce-Gorvel V Delrue RM Conde-Álvarez R Muñoz PM Lopez-Goñi I Iriarte M Marín CM Weintraub A Widmalm G Zygmunt M Letesson JJ Gorvel JP Blasco JM Moriyón I Brucellosis vaccines: assessment of *Brucella melitensis* lipopolysaccharide rough mutants defective in core and O-polysaccharide synthesis and export PLoS One 2008 3 e2760 10.1371/journal.pone.0002760 18648644 PMC2453230

[B10] Mancilla M Lopez-Goñi I Moriyón I Zarraga AM Genomic Island 2 is an unstable genetic element contributing to *Brucella* lipopolysaccharide spontaneous smooth-to-rough dissociation J Bacteriol 2010 192 6346 6351 10.1128/JB.00838-10 20952568 PMC3008527

[B11] Vizcaíno N Caro-Hernandez P Cloeckaert A Fernandez-Lago L DNA polymorphism in the omp25/omp31 family of *Brucella* spp.: identification of a 1.7-kb inversion in *Brucella cetaceae* and of a 15.1-kb genomic island, absent from *Brucella ovis*, related to the synthesis of smooth lipopolysaccharide Microbes Infect 2004 6 821 834 10.1016/j.micinf.2004.04.009 15374004

[B12] García-Yoldi D Marín CM Lopez-Goñi I Restriction site polymorphisms in the genes encoding new members of group 3 outer membrane protein family of *Brucella* spp FEMS Microbiol Lett 2005 245 79 84 10.1016/j.femsle.2005.02.026 15796983

[B13] Conde-Álvarez R Arce-Gorvel V Iriarte M Mancek-Keber M Barquero-Calvo E Palacios-Chaves L Chacón-Díaz C Chaves-Olarte E Martirosyan A Von-Bargen K Grilló MJ Jerala R Brandenburg K Llobet E Bengoechea JA Moreno E Moriyón I Gorvel JP The lipopolysaccharide core of *Brucella abortus* acts as a shield against innate immunity recognition PLoS Pathog 2012 8 e1002675 10.1371/journal.ppat.1002675 22589715 PMC3349745

[B14] Mancilla M Marín CM Blasco JM Zarraga AM Lopez-Goñi I Moriyón I Spontaneous excision of the O-polysaccharide *wbkA* glycosyltranferase gene is a cause of dissociation of smooth to rough *Brucella* colonies J Bacteriol 2012 194 1860 1867 10.1128/JB.06561-11 22328663 PMC3318470

[B15] Turse JE Pei J Ficht TA Lipopolysaccharide-deficient *Brucella* variants arise spontaneously during infection Front Microbiol 2011 2 54 21833310 10.3389/fmicb.2011.00054 PMC3153030

[B16] Vemulapalli R McQuiston JR Schurig GG Sriranganathan N Halling SM Boyle SM Identification of an IS711 element interrupting the *wboA* gene of *Brucella abortus* vaccine strain RB51 and a PCR assay to distinguish strain RB51 from other *Brucella* species and strains Clin Diagn Lab Immunol 1999 6 760 764 10473532 10.1128/cdli.6.5.760-764.1999 PMC95769

[B17] Zygmunt MS Blasco JM Letesson JJ Cloeckaert A Moriyón I DNA polymorphism analysis of *Brucella* lipopolysaccharide genes reveals marked differences in O-polysaccharide biosynthetic genes between smooth and rough *Brucella* species and novel species-specific markers BMC Microbiol 2009 9 92 10.1186/1471-2180-9-92 19439075 PMC2698832

[B18] DelVecchio VG Kapatral V Redkar RJ Patra G Mujer C Los T Ivanova N Anderson I Bhattacharyya A Lykidis A Reznik G Jablonski L Larsen N D’Souza M Bernal A Mazur M Goltsman E Selkov E Elzer PH Hagius S O’Callaghan D Letesson JJ Haselkorn R Kyrpides N Overbeek R The genome sequence of the facultative intracellular pathogen *Brucella melitensis* Proc Natl Acad Sci USA 2002 99 443 448 10.1073/pnas.221575398 11756688 PMC117579

[B19] National Center for Biotechnology Information/Basic Local Aligment Search Tool (NCBI/BLAST) [http://www.ncbi.nlm.nih.gov/BLAST]

[B20] European Molecular Biology Laboratory-European Bioinformatics Institute (EMBI-EBI) [http://www.ebi.ac.uk/Tools/clustalo]

[B21] National Center for Biotechnology Information/Primer-BLAST (NCBI/Primer-BLAST) [http://www.ncbi.nlm.nih.gov/tools/primer-blast/]

[B22] Wilson K Preparation of genomic DNA from bacteria Curr Protoc Mol Biol 1997 2.4.1 2.4.5 10.1002/0471142727.mb0204s56 18265184

[B23] Ocampo-Sosa AA Aguero-Balbin J Garcia-Lobo JM Development of a new PCR assay to identify *Brucella abortus* biovars 5, 6 and 9 and the new subgroup 3b of biovar 3 Vet Microbiol 2005 110 41 51 10.1016/j.vetmic.2005.06.007 16029934

[B24] Bosseray N *Brucella melitensis* Rev.1 living attenuated vaccine: stability of markers, residual virulence and immunogenicity in mice Biologicals 1991 19 355 363 10.1016/S1045-1056(05)80025-9 1797046

[B25] Grilló MJ Bosseray N Blasco JM In vitro markers and biological activity in mice of seed lot strains and commercial *Brucella melitensis* Rev 1 and *Brucella abortus* B19 vaccines Biologicals 2000 28 119 127 10.1006/biol.2000.0249 10885618

[B26] Conde-Álvarez R Grilló MJ Salcedo SP De-Miguel MJ Fugier E Gorvel JP Moriyón I Iriarte M Synthesis of phosphatidylcholine, a typical eukaryotic phospholipid, is necessary for full virulence of the intracellular bacterial parasite *Brucella abortus* Cell Microbiol 2006 8 1322 1335 10.1111/j.1462-5822.2006.00712.x 16882035

[B27] Quandt J Hynes MF Versatile suicide vectors which allow direct selection for gene replacement in gram-negative bacteria Gene 1993 127 15 21 10.1016/0378-1119(93)90611-6 8486283

[B28] Simon R Priefer U Pehle A A broad host range mobilization system for the *in vitro* genetic engineering: transposon mutagenesis in gram negative bacteria Biotechnology 1983 1 784 890 10.1038/nbt1183-784

[B29] Rajashekara G Covert J Petersen E Eskra L Splitter G Genomic island 2 of *Brucella melitensis* is a major virulence determinant: Functional analyses of genomic islands J Bacteriol 2008 190 6243 6252 10.1128/JB.00520-08 18641138 PMC2546784

[B30] Oy Growth Curves Ab Ltd [http://www.bioscreen.fi]

[B31] Aragón V Díaz R Moreno E Moriyón I Characterization of *Brucella abortus* and *Brucella melitensis* native haptens as outer membrane O-type polysaccharides independent from the smooth lipopolysaccharide J Bacteriol 1996 178 1070 1079 8576040 10.1128/jb.178.4.1070-1079.1996 PMC177767

[B32] Grilló MJ Manterola L De-Miguel MJ Muñoz PM Blasco JM Moriyón I Lopez-Goñi I Increases of efficacy as vaccine against *Brucella abortus* infection in mice by simultaneous inoculation with avirulent smooth *bvrS*/*bvrR* and rough *wbkA* mutants Vaccine 2006 24 2910 2916 10.1016/j.vaccine.2005.12.038 16439039

[B33] OIE Bovine Brucellosis Manual of Diagnostic Tests and Vaccines for Terrestrial Animals 2009 OIE 1 35 Chapter 2.4.3

[B34] Grilló MJ Blasco JM Gorvel JP Moriyón I Moreno E What have we learned from brucellosis in the mouse model? Vet Res 2012 43 29 10.1186/1297-9716-43-29 22500859 PMC3410789

[B35] Gerhardt P The nutrition of Brucellae Bacteriol Rev 1958 22 91 98 10.1128/br.22.2.81-98.1958 PMC180938 13546130

[B36] Cloeckaert A Grayon M Grepinet O Identification of Brucella melitensis vaccine strain Rev.1 by PCR-RFLP based on a mutation in the rpsL gene Vaccine 2002 20 2546 2550 10.1016/S0264-410X(02)00159-7 12057611

[B37] Maisnier-Patin S Berg OG Liljas L Andersson DI Compensatory adaptation to the deleterious effect of antibiotic resistance in *Salmonella* Typhimurium Mol Microbiol 2002 46 355 366 10.1046/j.1365-2958.2002.03173.x 12406214

[B38] Manson JM Gilmore MS Pathogenicity island integrase cross-talk: a potential new tool for virulence modulation Mol Microbiol 2006 61 555 559 10.1111/j.1365-2958.2006.05262.x 16879637

