# Supplementary material for: Deletion of the GI-2 integrase and the wbkA flanking transposase improves the stability of Brucella melitensis Rev 1 vaccine
Source: Vet Res. 2013 Oct 31;44(1):105. doi: 10.1186/1297-9716-44-105 (PMC4176087; doi:10.1186/1297-9716-44-105)
Supplement: Additional file 1 — E. coli strains and plasmids used. List, characteristics and source/reference of E. coli strains and plasmids used. [file 1297-9716-44-105-S1.docx]

| Strain/plasmid | Characteristics | Source/ reference |
| --- | --- | --- |
| TOP10F’ | F - *lacI*q Tn *10* (TetR) mcrA D(*mrr-hsdRMS-mcrBC*) 80*lac*ZDM15 D*lacX74 recA1alaD139* D (*ara-leu*)*7697galU galK rpsL endA1 nupG* | Invitrogen |
| OmniMAX 2-t1 | F′ [*proAB*+ *lacIq* *lacZ*∆M15 Tn10(TetR) ∆(*ccdAB*)] mcrA ∆(*mrr-hsdRMS-mcrBC*) φ80(*lacZ*)∆M15 ∆(*lacZYA-argF*) U169 *endA1* *recA1* supE44 thi-1 *gyrA*96 *relA1* *tonA panD* | Invitrogen |
| S17-1λ*pir* | Mating strain with plasmid RP4 inserted into the chromosome | [28] |
| pCR2.1 TOPO | Cloning plasmid. Km and Amp resistance | Invitrogen |
| pJQK | Suicide vector for mutagenesis by allele replacement. Km resistance | [27] |
| pDONR221 | Cloning plasmid Gateway™ technology. Km resistance | Invitrogen |
| pMR10 | Destination vector, Gateway™ technology. Km and Ch resistance | [39] |
| pMM14 | Complementation vector, pMR10 derivative containing a copy of *wbkA* gene. Km and Ch resistance | [14] |
| pMM20 | pCR2.1 derivative containing an insert generated by PCR overlap to delete ORFs BMEI1398-1400 (IS*Bm1*) | This study |
| pMM22 | Mutator plasmid, pJQK derivative containing *Bam*HI-*Not*I fragment from pMM20 | This study |
| pMM55 | pCR2.1 derivative containing a fragment generated by PCR overlap with mutant allele of *int* (BMEI1012) | This study |
| pMM56 | Mutator plasmid, pJQK derivative containing *Bam*HI-*Xba*I fragment from pMM55 | This study |
| pMM75 | pDONR221 derivative containing glycosyltransferases genes *wboA*-*wboB* from *B. melitensis* 16M. Km resistance | This study |
| pMM76 | Complementation vector, pMR10 derivative with *att* fragment from pMM75 containing *wboAB* genes. Km and Ch resistance | This study |
| pBGI-997-99c | Complementation vector, pBBR1mcs-4 derivative containing ORFs BMEI0997-999 from *B. melitensis* 16M. Amp resistance | [29] |

Km: kanamycin; Amp: ampicillin; Ch: chloramphenicol.
